# A New Endogenous Overexpression System of Multidrug Transporters of *Candida albicans* Suitable for Structural and Functional Studies

**DOI:** 10.3389/fmicb.2016.00261

**Published:** 2016-03-03

**Authors:** Atanu Banerjee, Nitesh K. Khandelwal, Dominique Sanglard, Rajendra Prasad

**Affiliations:** ^1^Membrane Biology Laboratory, School of Life Sciences, Jawaharlal Nehru UniversityNew Delhi, India; ^2^Institute of Microbiology, University of Lausanne and University Hospital CenterLausanne, Switzerland; ^3^Amity Institute of Integrative Sciences and Health and Amity Institute of Biotechnology, Amity University HaryanaGurgaon, India

**Keywords:** *Candida albicans*, multidrug transporters, endogenous overexpression system, Cdr1p, Mdr1p, TAC1 GOF

## Abstract

Fungal pathogens have a robust array of multidrug transporters which aid in active expulsion of drugs and xenobiotics to help them evade toxic effects of drugs. Thus, these transporters impose a major impediment to effective chemotherapy. Although the *Saccharomyces cerevisiae* strain AD1-8u^−^ has catered well to the need of an overexpression system to study drug transport by multidrug transporters of *Candida albicans*, artifacts associated with a heterologous system could not be excluded. To avoid the issue, we exploited a azole-resistant clinical isolate of *C. albicans* to develop a new system devoid of three major multidrug transporters (Cdr1p, Cdr2p, and Mdr1p) for the overexpression of multidrug transporters under native hyperactive *CDR1* promoter due to gain of function (GOF) mutation in *TAC1*. The study deals with overexpression and functional characterization of representatives of two major classes of multidrug transporters, Cdr1p and Mdr1p, to prove the functionality of this newly developed endogenous expression system. Expression of native Cdr1 and Mdr1 protein in *C. albicans* cells was confirmed by confocal microscopy and immunodetection and resulted in increased resistance to the putative substrates as compared to control. The system was further validated by overexpressing a few key mutant variants of Cdr1p and Mdr1p. Together, our data confirms the utility of new endogenous overexpression system which is devoid of artifactual factors as most suited for functional characterization of multidrug transporter proteins of *C. albicans*.

## Introduction

*C. albicans*, an otherwise commensal of the human microbiome, can become causative agent of superficial as well as life-threatening systemic infections (Staib et al., 2000). Prolonged use of antifungals, primarily azoles, has led to the emergence of Multidrug resistance (MDR). Although MDR is a multi-factorial response, the overexpression of membrane pumps such as ABC (ATP-binding Cassette transporters) and MFS (Major-Facilitator Superfamily) transporters are the prime actors in contributing to the development of tolerance to drugs (White, 1997; Rogers and Barker, 2003).

Heterologous overexpression of MDR pump proteins of *C. albicans* in *Saccharomyces cerevisiae* has been widely used as a novel tool for the structure-function analyses of these transporters. Such studies unpinned crucial molecular details of drug efflux, protein trafficking and transport cycle (Lamping et al., 2007; Pasrija et al., 2007; Rawal et al., 2013; Shah et al., 2015a,b). Furthermore, this system also provided novel insights regarding array of substrates recognized by these transporters (Puri et al., 2010) and critical amino acids involved in the substrate and inhibitor recognition (Saini et al., 2005; Pasrija et al., 2007; Niimi et al., 2012; Rawal et al., 2013; Nim et al., 2014). Together, the afore-mentioned applications of the heterologous system has helped in the development of therapeutic inhibitors and modulators of efflux pumps (Hayama et al., 2012; Maurya et al., 2013).

In spite of their insightful contributions, artifactual concerns associated with a heterologous background could not be neglected. Differences pertaining to the membrane components, primarily lipids could influence the insertion and proper association of the foreign transporters within the membrane compartment and thus might have an impact on the structural as well as functional properties of these proteins (Opekarova and Tanner, 2003). Moreover, the alternative codon usage of *C. albicans* requires mutational corrections of some codons when specific *C. albicans* genes need to be expressed in *S. cerevisiae* (Santos and Tuite, 1995).

To circumvent artifactual effects of a heterologous system and also to avoid the need of codon corrections, we have developed here an endogenous model system for the overexpression of clinically relevant multidrug transporters of *C. albicans*. For this, we used an azole-resistant clinical isolate of *C. albicans* where genes encoding major drug transporters *CDR1, CDR2*, and *MDR1* were deleted. This strain, although deleted of its major MDR attributes, still contains a gain of function (GOF) mutation in *TAC1* transcription factor which is responsible for a constitutive overexpression of *CDR1* (Znaidi et al., 2007). A GFP tagged variant of Cdr1p was generated and integrated at its native chromosomal locus. It was observed that the overexpressed protein was properly localized to the plasma membrane and could confer drug resistance to the strain. The study confirms that this overexpression system is not only suitable for the expression of *CDR1* but equally suitable for non-ABC transporter genes such as *MDR1*.

## Materials and methods

### Bacterial and yeast strains

Yeast strains used in the study are listed in Table 1. Plasmids were maintained in *E. coli* Dh5α strain cultured in Leuria Bertani medium (HiMedia Laboratories, Mumbai, India) to which ampicillin (Amresco, Solon, USA) was added at a final concentration of 0.1 mg/ml. The yeast strains were cultured in either YEPD broth or on YEPD agar plates. YEPD broth was procured from HiMedia Laboratories, Mumbai, India. For selection of yeast transformants after integration, SD-Ura^−^ drop out medium with 2% agar was used. SD-Ura^−^ drop out medium comprised of 0.67% YNB medium without amino acids (Difco, Detroit, MI), 0.2% Ura^−^ dropout mix and 2% glucose (Fisher-Scientific, Mumbai, India).

**Table 1:** **List of yeast strains used in the study**.

**Strain**	**Genotype/Description**	**Parent strain**	**Source/Reference**
STY31	*cdr2*AΔ::FRT/*cdr2*BΔ::FRT *cdr1*AΔ::FRT/*cdr1*BΔ::FRT	5674	Tsao et al., 2009
DSY4680	*cdr2*AΔ::FRT/*cdr2*BΔ::FRT *cdr1*AΔ::FRT/*cdr1*BΔ::FRT *ura3*Δ::FRT/*ura3*Δ::FRT	STY31	This study
DSY4684	*cdr2*AΔ::FRT/*cdr2*BΔ::FRT *cdr1*AΔ::FRT/*cdr1*BΔ::FRT *ura3*Δ::FRT/*ura3*Δ::FRT *mdr1*Δ::hisG/*mdr1*Δ::hisG	DSY4680	This study
DSY4687-5	*cdr2*AΔ::FRT/*cdr2*BΔ::FRT *cdr1*AΔ::FRT::*CDR1*- GFP::*URA3* /*cdr1*BΔ::FRT *ura3*Δ::FRT/*ura3*Δ::FRT *mdr1*Δ::hisG/*mdr1*Δ::hisG	DSY4684	This study
ANY-L529A	*cdr2*AΔ::FRT/*cdr2*BΔ::FRT *cdr1*AΔ::FRT::*CDR1*^L529A^ -GFP::*URA3* / *cdr1*BΔ::FRT *ura3*Δ::FRT/*ura3*Δ::FRT/ *mdr1*Δ::hisG/ *mdr1*Δ::hisG	DSY4687-5	This study
ANY-V532A	*cdr2*AΔ::FRT/*cdr2*BΔ::FRT *cdr1*AΔ::FRT::*CDR1*^V532A^ -GFP::*URA3* / *cdr1*BΔ::FRT *ura3*Δ::FRT/*ura3*Δ::FRT/ *mdr1*Δ::hisG/ *mdr1*Δ::hisG	DSY4687-5	This study
ANY-C1294A	*cdr2*AΔ::FRT/*cdr2*BΔ::FRT *cdr1*AΔ::FRT::*CDR1*^C1294A^ -GFP::*URA3* / *cdr1*BΔ::FRT *ura3*Δ::FRT/*ura3*Δ::FRT/ *mdr1*Δ::hisG/ *mdr1*Δ::hisG	DSY4687-5	This study
ANY-MDR1-GFP	*cdr2*AΔ::FRT/*cdr2*BΔ::FRT *cdr1*AΔ::FRT::*MDR1*- GFP::*URA3*)/*cdr1*BΔ::FRT *ura3*Δ::FRT/*ura3*Δ::FRT *mdr1*Δ::hisG/*mdr1*Δ::hisG	DSY4684	This study
ANY-W248A	*cdr2*AΔ::FRT/*cdr2*BΔ::FRT *cdr1*AΔ::FRT::*MDR1*^W248A^_–_ GFP::*URA3* /*cdr1*BΔ::FRT *ura3*Δ::FRT/*ura3*Δ::FRT *mdr1*Δ::hisG/*mdr1*Δ::hisG	ANY-MDR1-GFP	This study

### Materials

Itraconazole (ITC), Clotrimazole (CTR), Ketoconazole (KTZ), Miconazole (MCZ) and Voriconazole (VOR), Anisomycin (ANI), Rhodamine 6G (R6G), 4-nitroquinoline 1-oxide (NQO), Adenosine triphosphate (ATP), Oligomycin (OM), DL-Dithiothreitol (DTT), Sorbitol, Phenylmethanesulfonyl fluoride (PMSF), p-Tosyl-L-lysine chloromethyl ketone (TLCK), and Tosyl phenylalanyl chloromethyl ketone (TPCK) were procured from Sigma Chemical Co. (St. Louis, MO). Protease inhibitors leupeptin, aprotinin, Pepstatin were obtained from G-biosciences, MO, USA). Fluconazole (FLC) was a generous gift from Ranbaxy Laboratories, India. Oligonucleotides were procured from Sigma Genosys, India and are listed in Table S2. Anti-GFP monoclonal antibody was purchased from Santa Cruz Biotechnology Inc. (Texas, USA). All other routine chemicals were purchased from Fisher-scientific, Mumbai, India.

### Plasmid constructions

The homologous expression system was constructed based on Clp10 (Murad et al., 2000). The CDR1 terminator (CDR1ter) (starting 21 nt downstream of the *CDR1* stop codon) was first cloned as a 519 bp NotI-SacI fragment into Clp10 to obtain pDS1859 (Table S1). The NotI-SacI fragment CDR1ter was obtained by PCR using *C. albicans* SC5314 DNA as template with primers CDR1ter_SacI and CDR1Ter_NotI. Next, pDS1859 was used to insert a modified CDR1 promoter (−1222 bp with respect to first ATG codon) in which a natural SpeI site was destroyed. This was accomplished by a two-step fusion PCR using a first fragment obtained with primers CDR1-Apa and SpeKO3 and a second fragment obtained with primers CDR1-SpeI and SpeKO5. The two purified PCR fragments were sewed by PCR using external primers CDR1-Apa and CDR1-SpeI. The obtained fragment was cloned in pDS1859 using ApaI and SpeI restriction sites to result in pDS1866. Next, the *S. cerevisiae PDR5* terminator was inserted as a SpeI-NheI fragment from pPSCDR1-GFP into pDS1866 to result in pDS1869. Finally the CDR1-GFP fusion from pPSCDR1-GFP was cloned as a SpeI fragment into pDS1869 to result in pDS1874.

For cloning of MDR1-GFP, the MDR1-GFP fusion from pPSMDR1-GFP was obtained by SpeI digestion. The fragment was next cloned in pDS1874 to replace CDR1-GFP, thus resulting in pAN-MDR1. The BglII site present in *MDR1* cloned in pAN-MDR1 was removed by site directed mutagenesis.

### Yeast strain constructions

The *C. albicans* strain STY31 (a kind gift from M. Raymond, Montreal) that lacks *CDR1* and *CDR2* (Tsao et al., 2009) was used as a starting strain for the deletion of *MDR1*. First, *ura3* alleles were inactivated sequentially using pSFSU1 (Coste et al., 2006) in STY31 to obtain DSY4680 after sequential *ura3* inactivation and recycling of the *SAT1* dominant marker. DSY4680 was used to inactivate both *MDR1* alleles with pDS287 as described (Sanglard et al., 1996). The resulting strain lacking *CDR1, CDR2*, and *MDR1* was named DSY4684. This strain contains the *TAC1* GOF N972D (Znaidi et al., 2007; Tsao et al., 2009). CDR1-GFP expression could be obtained in this strain by transformation with pDS1874 digested by BglII to facilitate integration in the *CDR1* promoter. MDR1-GFP expression was obtained in DSY4684 by transformation with pAN-MDR1 digested by BglII to facilitate integration in the *CDR1* promoter.

### Site-directed mutagenesis

Site-directed mutagenesis was performed by using Quick-Change site directed mutagenesis kit (Agilent Technologies, USA). The mutations were incorporated in pDS1874 and pAN-MDR1 plasmids according to the manufacturer's instructions and the nucleotide changes were confirmed by sequencing. Confirmed plasmid clones were used for transformation of DSY4684 using electroporation after linearization with BglII as described previously (Puri et al., 2011).

### Drug susceptibility assays

The susceptibilities of the different yeast strains to different drugs were tested using broth microdilution and serial dilution spot assays as described previously (Shah et al., 2015b).

### Agar drug diffusion assay

Overnight grown cultures were diluted to an OD_600_ of 0.01 in YEPD agar and poured onto petri plate. Whatman paper filter discs containing the xenobiotics were placed on top of the YEPD agar plate and incubated for 48 h at 30°C. Amount of xenobiotics used are mentioned in the figure legends. For the chemosensitization assay, FLC at ¼ MIC_FLC_ was added to the YEPD agar containing 10^5^ cells and the inhibitor containing disc was placed on to the plate.

### Immunodetection of GFP tagged proteins

Plasma membrane fractions used for immunodetection of GFP tagged proteins were prepared as described previously (Shukla et al., 2003) with minor modifications. Here, cells used for preparation for PM fractions were harvested after 8 h of growth. 50 micrograms of total PM proteins were used for immunodetection with HRP-labeled anti-GFP antibody at 1:5000 dilution. Protein bands were detected by BIO-RAD ChemiDoc XRS+ system following reaction with Clarity Western ECL blotting substrate (Bio-Rad).

### Immunodetection of Cdr1

*C. albicans* total protein extracts were prepared as described (Coste et al., 2007). Same protein quantites were loaded for gel eletrophoresis and Cdr1 immunodection was performed as earlier described by chemiluminescence (ECL kit, Amersham, Bioscience) using polyclonal rabbit anti-Cdr1 antibody and an anti-rabbit-HRP labeled secondary antibody. Chemiluminescence was recorded with a ImageQuant LAS 4000 mini CCD camera (GE Healthcare Bio-Sciences).

### Fluorescence and confocal microscopy

Cells were harvested at the requisite time points from a secondary culture, washed and viewed under Nickon Ti90 fluorescence microscope to visualize the GFP-tagged protein. For Confocal microscopy, 8 h grown cells were harvested and imaged using Olympus FluoView™FV1000 laser confocal microscope (PA, USA) or Nickon Eclipse Ti E laser confocal microscope with 100X oil immersion objective lens.

### Substrate transport assays

R6G efflux of the strains was determined as described previously (Shah et al., 2015b) with minor modifications. Briefly, overnight grown cultures were used to set an OD of 0.4 in 15 ml of YEPD medium. The cultures were then grown for 8 h. Cells were harvested, washed and resuspended in 1.5 ml Phosphate buffered saline (PBS) to make a final OD of 10. The cell suspension was then grown in the presence of R6G at 10 μM final concentration for 2 h. Cells were then harvested, washed and resuspended in 1.5 ml PBS containing 2% glucose. After 45 min, cells were pelleted and 1 ml of the supernatant was used to measure the absorbance at 530 nm. For NR accumulation, 8 h grown cultures were used to set 0.2 OD in 1 ml of PBS containing 2% glucose. The suspension was incubated with 7 μM final concentration of NR. After 45 min, the cells were harvested, washed with PBS and used and NR accumulation was monitored by flow cytometry using a FACSort flow cytometer (Becton–Dickinson Immunocytometry Systems, San Jose, CA). CellQuest software (Becton-Dickinson Immunocytometry Systems, San Jose, CA) was used for data analysis. For [^3^H] FLC (specific activity 2.8 Ci/mmol) accumulation assay, log phase cells were harvested, washed with PBS (Phosphate buffered saline, pH 7.4) and finally resuspended as 5% cell suspension. After de-energization at 30°C for 2 h, 0.25 ml of 5% cell suspension (in PBS with 2% glucose) was incubated with 100 nM final concentration of [^3^H] FLC. A 100 μl aliquot was taken after 60 min of incubation at 30°C filtered through a 0.45 μm cellulose nitrate filter using millipore manifold filtration assembly and washed twice with PBS. The filter discs were dried under a lamp and then immersed in liquid scintillation cocktail mixture (Cocktail O, SRL, India) to measure the accumulated radioactivity using liquid scintillation counter (Tri-Carb 2900TR Liquid Scintillation Analyzer; Packard).

### Oligomycin-sensitive ATPase assay

ATPase activity of the strains was monitored by the oligomycin-sensitive release of inorganic phosphate as described previously (Shah et al., 2015b). Briefly, 10 μg total PM protein was used to determine the ATPase activity in an *in vitro* assay. 5 mM ATP was used in the reaction mixture which was terminated after 30 min of incubation at 30°C by addition of 1 ml of stop solution which contained 0.5% SDS, 0.5% ammonium molybdate and 2% H_2_SO_4_. 10 μl of 10% ascorbic acid was added to the solution and further incubated for 30 min for development of color. Absorbance of the solution was measured at 880 nm to assess the P_i_ release.

### Statistical analyses

Data is represented as mean ± SD. The statistical analyses were performed using Student's *T*-Test (http://www.studentsttest.com/). Differences were considered statistically significant when *p* < 0.05 (^*^ signifies *p* < 0.05, ^**^ signifies *p* ≤ 0.01 and ^***^ signifies *p* ≤ 0.001).

## Results

### Expression of Cdr1-GFP in a MDR depleted strain of *C. albicans*

**Figure 1:**
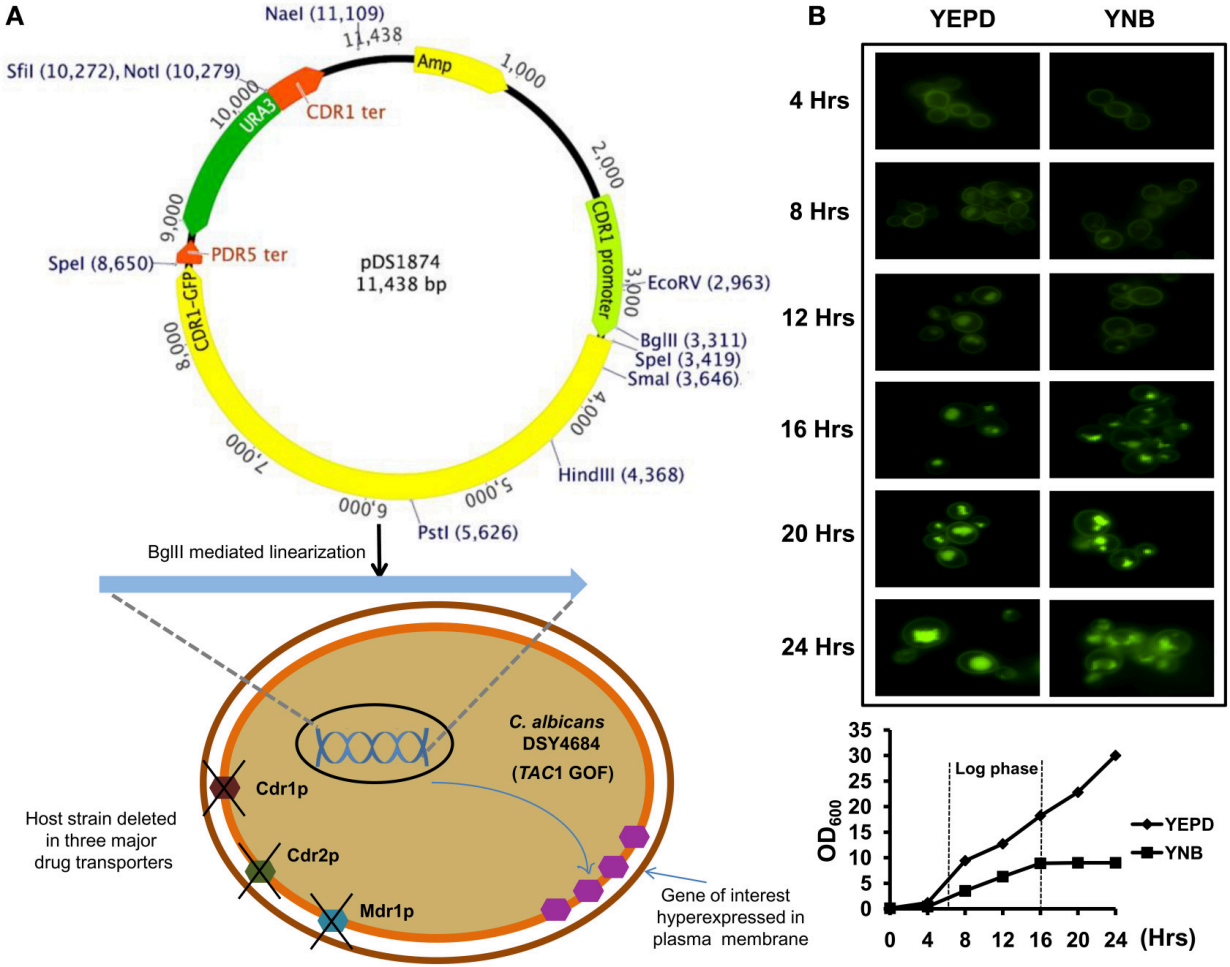
**(A)** Schematic representation of the *C. albicans* transporter overexpression system. The system contains the promoter and terminator regions of *CDR1* and a *ura3* selection marker. The SpeI cloning site in the plasmid can be used to insert the gene of interest. A *S. cerevisiae PDR5* terminator is situated downstream of the SpeI site. The plasmid can be linearized by BglII digestion and integrated into the host strain DSY4684 which is devoid of three major MDR pumps of *C. albicans* (*CDR1, CDR2*, and *MDR1*). The host strain also has a GOF mutation in the *TAC1* transcription factor as a result of which the cloned ORF is overexpressed under the native *CDR1* promoter. **(B)** Time course fluorescence microscopy of DSY4687-5 strain (overexpressing Cdr1-GFP) grown in YEPD and YNB medium. Lower panel shows growth curve of the strain in the two mentioned medium at the time points used for microscopic analyses.

**Figure 2:**
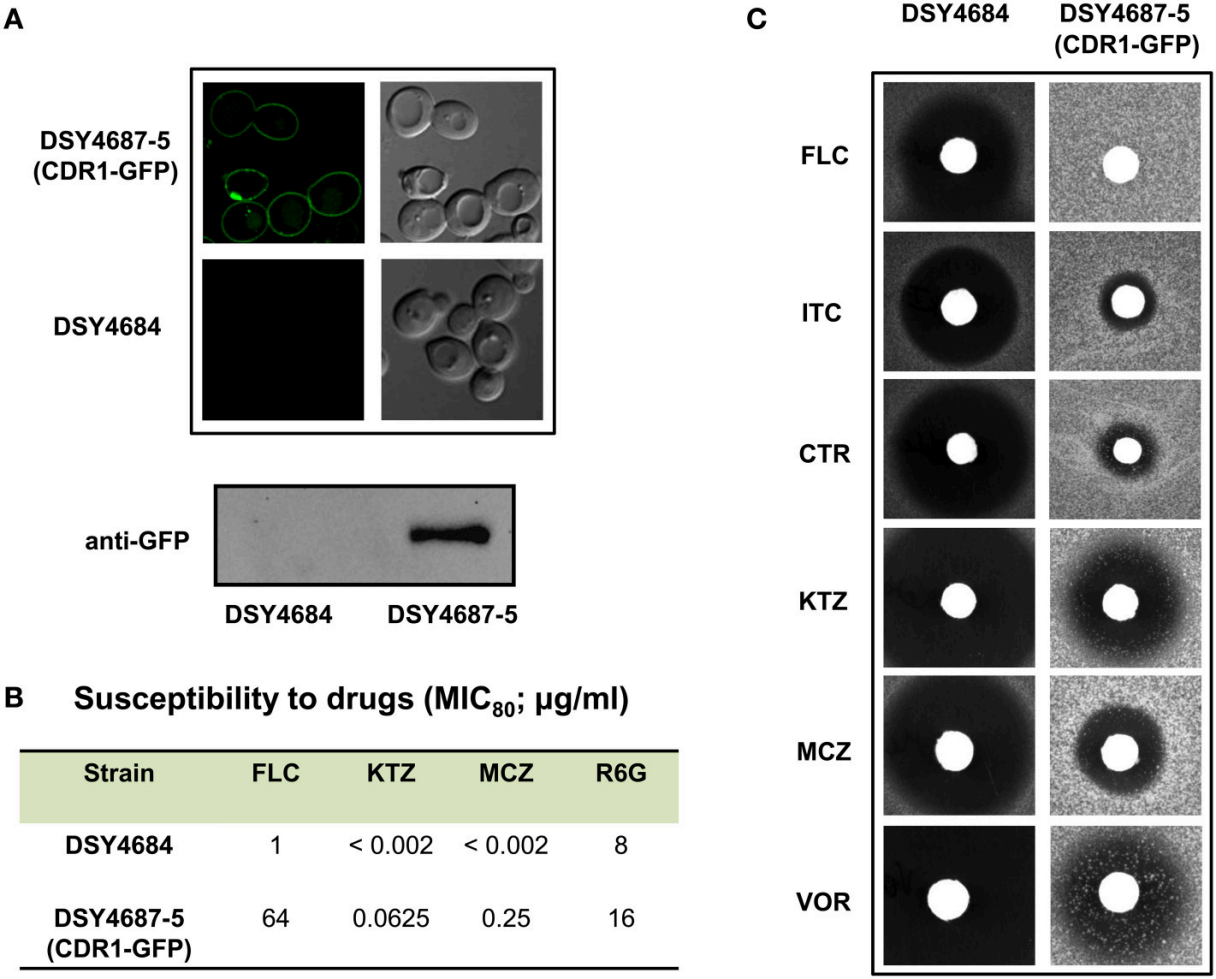
**(A)** Confocal microscopy images of DSY4684 and DSY4687-5 strains (Upper panel) and immunoblot analyses of the PM fractions (50 μg total protein) with anti-GFP monoclonal antibody (Lower panel). **(B)** MIC_80_ values of DSY4684 and DSY4687-5 cells for Fluconzole (FLC), Ketoconazole (KTZ), Miconazole (MCZ) and Rhodamine 6G (R6G). **(C)** Agar drug-diffusion assays of DSY4684 and DSY4687-5 cells. Susceptibility to the following azoles were tested: Fluconazole (FLC; 10 μg), Itraconazole (ITC; 1 μg), Clotrimazole (CTR; 0.5 μg), Ketoconazole (KTZ; 0.2 μg), Miconazole (MCZ; 0.2 μg) and Voriconazole (VOR; 1 μg).

An AR isolate with a GOF mutation in *TAC1* was further derivatized by deleting its major MDR pump candidates, Cdr1p, Cdr2p, and Mdr1p as well as *ura3* for selection. This strain was designated as DSY4684. The *CDR1* gene with a C-terminal GFP tag was introduced at the native chromosomal locus in DSY4684 by homologous recombination using *ura3* as the selection marker. The integration of the CDR1-GFP chimeric construct was performed by single (BglII) digestion of pDS1874. Resulting strain was designated as DSY4687-5 (Figure 1A) and (Figure S1). Fluorescence microscopy at different growth time points revealed that Cdr1-GFP was overexpressed and correctly trafficked to the plasma membrane (PM) of DSY4687-5 (Figure 1B). Notably, the fluorescent intensity of Cdr1-GFP was the highest at 4 hours of growth and declined subsequently with time. A significant amount of fluorescence was observed within the vacuoles after 12 hours indicating that the overexpressed protein was being targeted to the vacuole for degradation. This expression trend was common in both defined (YNB) and enriched medium (YEPD). At 8 hours of growth, a considerable amount of the protein was still visible within the PM which was confirmed by confocal and immunoblot analyses of the PM fractions with an anti-GFP antibody (Figure 2A). Since the cellular yields were sufficient for functional analyses, all the subsequent studies were performed at this growth time point.

### Overexpressed Cdr1-GFP is functional

We evaluated the drug susceptibilities of DSY4687-5 and DSY4684 strains to assess the substrate transport ability of the overexpressed Cdr1-GFP. MIC_80_ values for the above mentioned strains against the four well known Cdr1p substrates including Fluconazole (FLC), Ketoconazole (KTZ), Miconazole (MCZ), and Rhodamine 6G (R6G) confirmed that these drugs were efficiently effluxed by the overexpressed protein (Figure 2B). Interestingly, overexpression of Cdr1p increased the resistance of DSY4684 to FLC and MCZ by more than 50- and 100-fold, respectively, whereas the fold change in resistance to KTZ and R6G was 30 and 2-fold, respectively. Agar drug-diffusion assays were employed to validate MIC_80_ results, which also confirmed the functionality of overexpressed Cdr1-GFP in DSY4684. It was evident from the difference in zone-of-inhibition between the tested strains that Cdr1-GFP could recognize and efflux its substrates (Figure 2C).

### Phenotypic analysis of selected mutants of overexpressed Cdr1p in *C. albicans*

**Figure 3:**
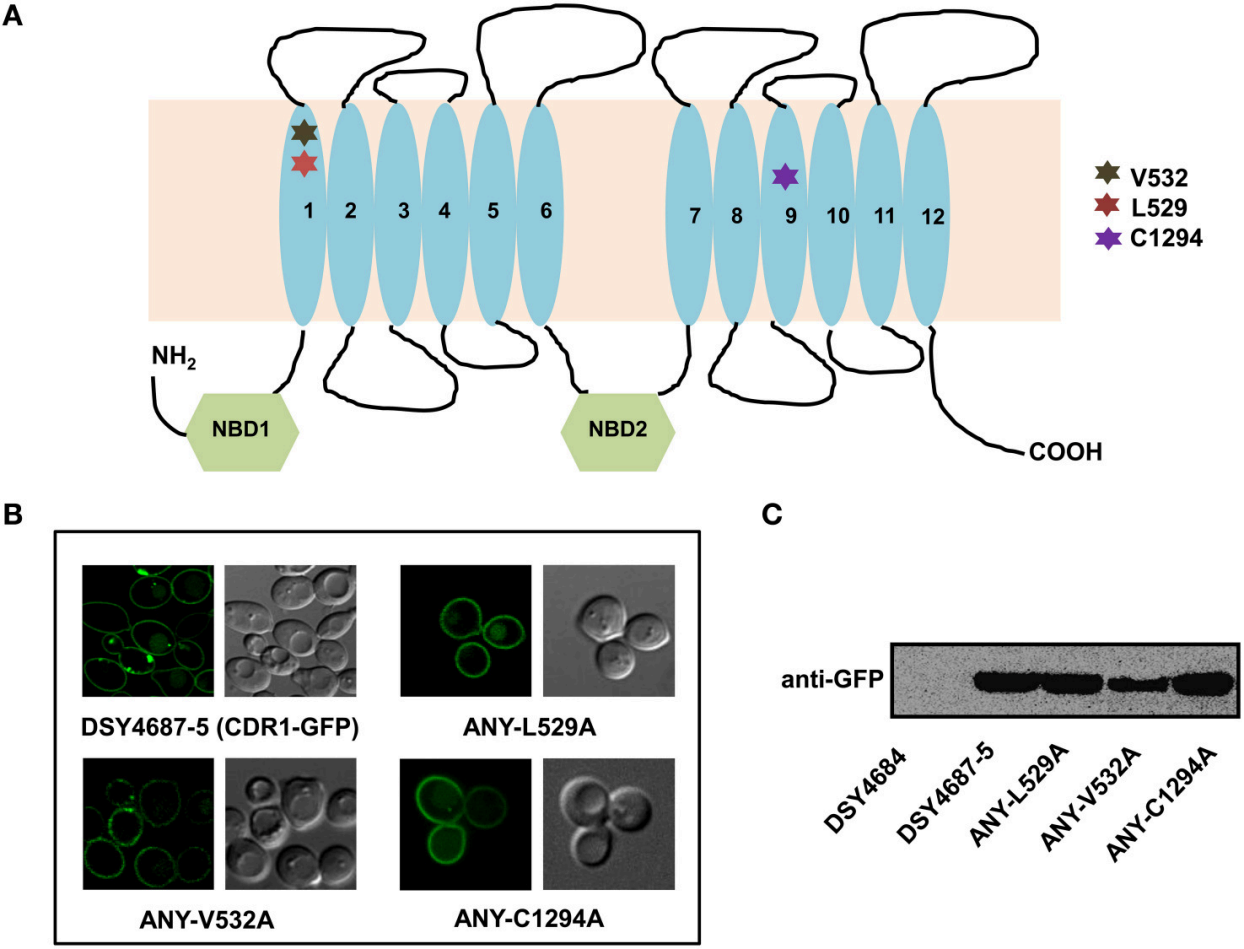
**(A)** Schematic diagram of the topology of Cdr1p with highlighted amino acids selected for replacement by alanine. **(B)** Confocal analyses of DSY4687-5 and the three constructed mutant variant strains, ANY-L529A, ANY-V532A, and ANY-C1294A. **(C)** Western analyses of the PM fractions (50 μg total protein) of the strains with anti-GFP monoclonal antibody.

In order to further validate the newly designed endogenous expression system, we subjected the Cdr1-GFP to site directed mutational analyses. For this, we selected three residues, L529, V532, and C1294 which were earlier shown to be crucial for the efflux of substrates of Cdr1p (Rawal et al., 2013). All the three mutant variants (L529A, V532A, and C1294A) overexpressed in *S. cerevisiae* rendered cells hypersusceptible to all the tested drugs. Substrate binding/transport and molecular modeling predicted that the three residues including L529, V532, and C1294 are part of the drug binding pocket (Rawal et al., 2013). Figure 3A highlights the positions of these residues within the transmembrane helices (TMHs). For instance, L529 and V532 are part of the TMH1 whereas C1294 is located within TMH9. Considering their importance, we recreated the alanine variants of these critical residues (L529A, V532A, and C1294A) and integrated them to native chromosomal location in DSY4684. The resulting strains were designated as ANY-L529A, ANY-V532A, and ANY-C1294A, respectively. Confocal microscopy and immunoblot analyses of the PM fractions with an anti-GFP antibody confirmed that all the mutant variants of Cdr1-GFP were targeted to the cell surface. However, the Cdr1^V532A^ variant was expressed at lower levels when compared to the wild type Cdr1 (DSY4687-5) and the other two variants (Figures 3B,C).

**Figure 4:**
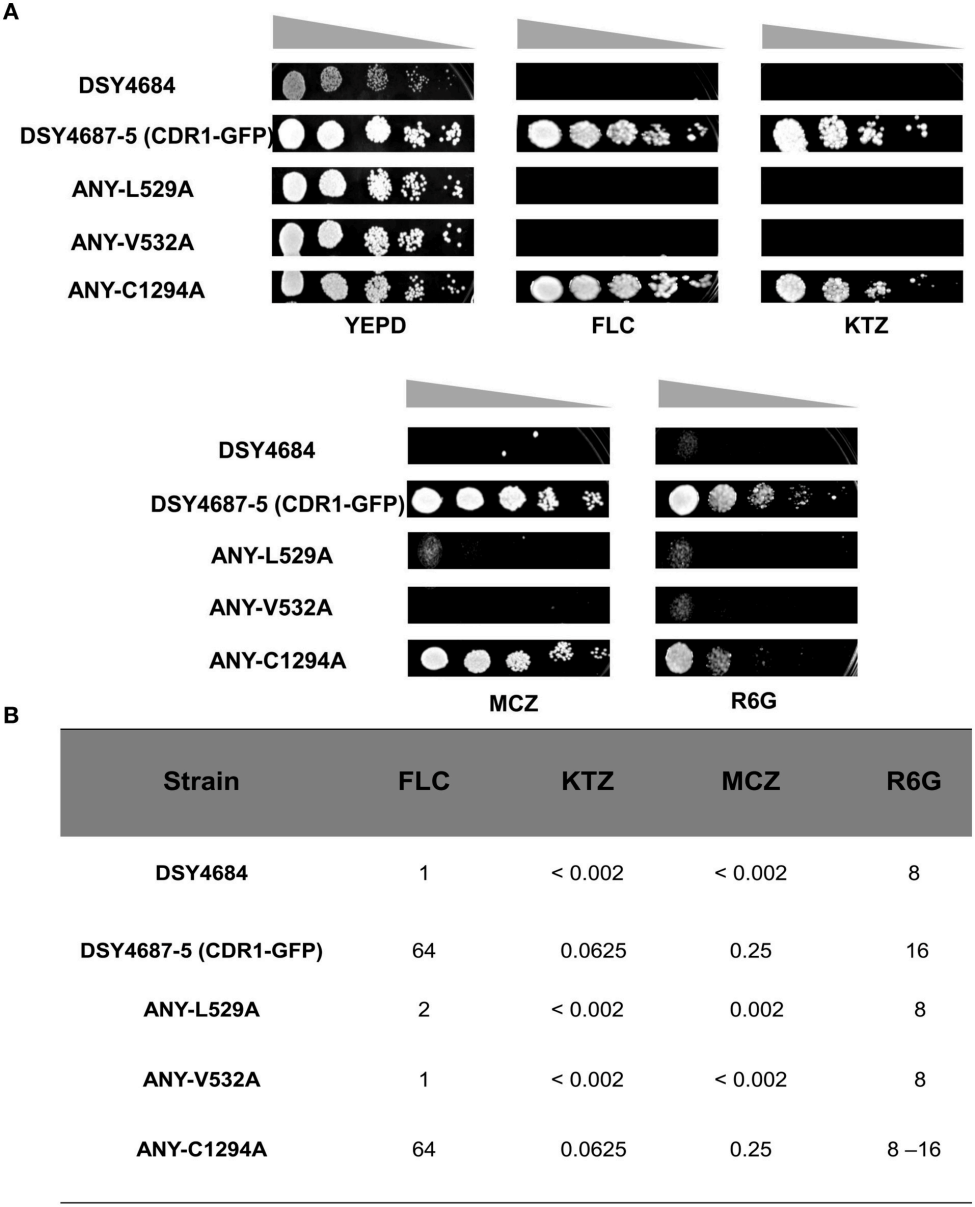
**(A)** Drug resistance profiles of DSY4684, DSY4687-5 and the mutant variants determined by serial dilution spot assays. For the assay, following drugs were used: FLC (8 μg/ml), KTZ (0.04 μg/ml), MCZ (0.04 μg/ml), R6G (8 μg/ml). **(B)** MIC_80_ values of DSY4687-5 and its mutant variants for FLC, KTC, MCZ, and R6G.

Serial dilution and liquid drug susceptibility assays were performed to assess the ability of the mutant variants to confer drug resistance. Expectedly, the results confirmed that the strains ANY-L529A and ANY-V532A were hypersusceptible to all the tested drugs corresponding to the results earlier observed in the overexpressing *S. cerevisiae* strain AD1-8u^−^. Interestingly, the Cdr1^C1294A^ variant showed only marginal susceptibility to R6G and remained resistant to other tested drugs (Figures 4A,B).

**Figure 5:**
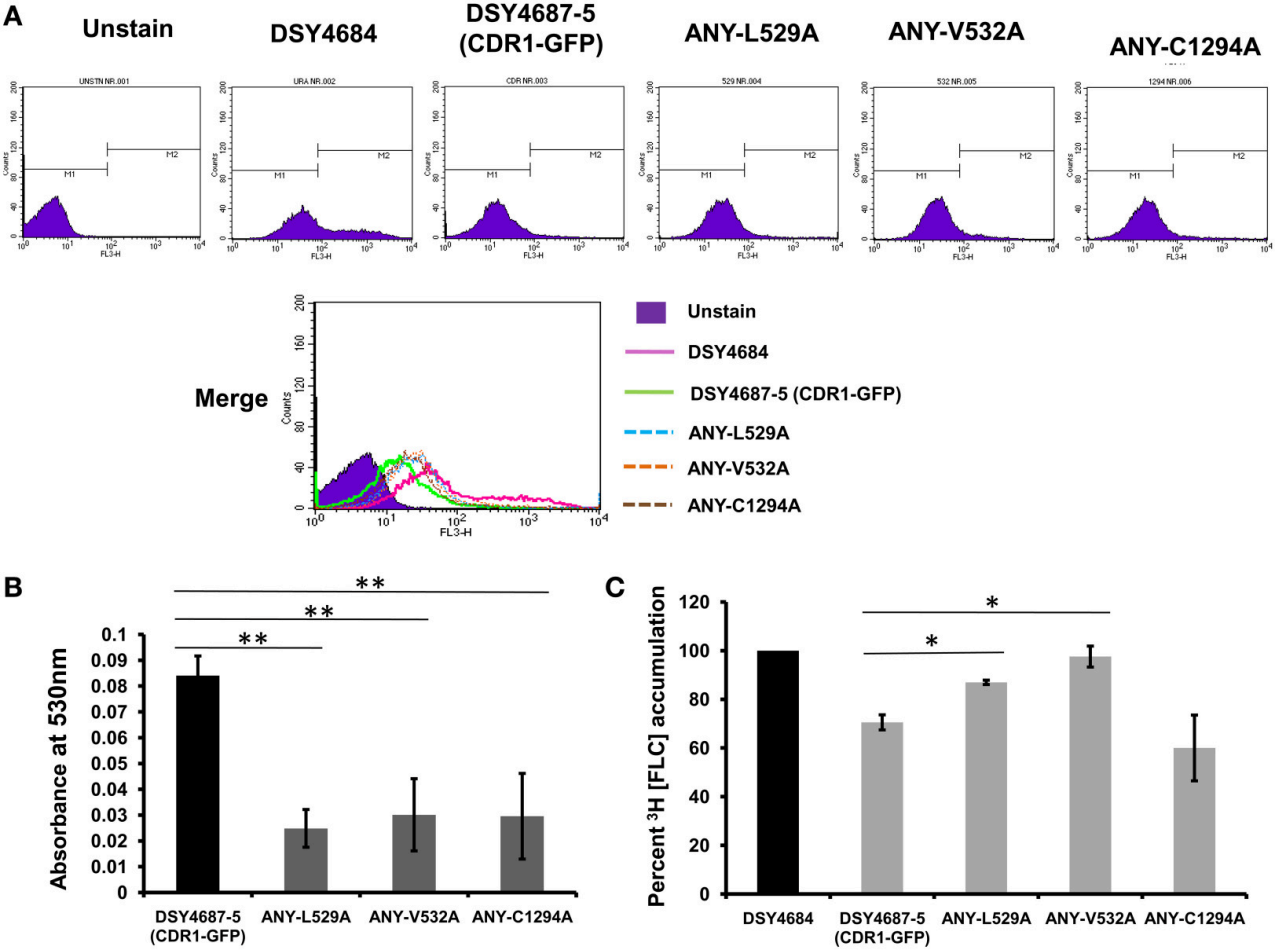
**Substrate transport assays of DSY4684, DSY4687-5 and the mutants. (A)** NR accumulation in cells overexpressing Cdr1-GFP and the mutant variants as determined by Flow cytometry analyses using FL3 filter **(B)** R6G efflux in cells overexpressing Cdr1-GFP and the mutant variants. Assay was performed as described in the experimental section. The host DSY4684 values were subtracted from each reading. **(C)** [^3^H] FLC accumulation in cells overexpressing Cdr1-GFP and the mutant variants with respect to DSY4684 (value kept at 100%). ^*^p < 0.05; ^**^p ≤ 0.01.

**Figure 6:**
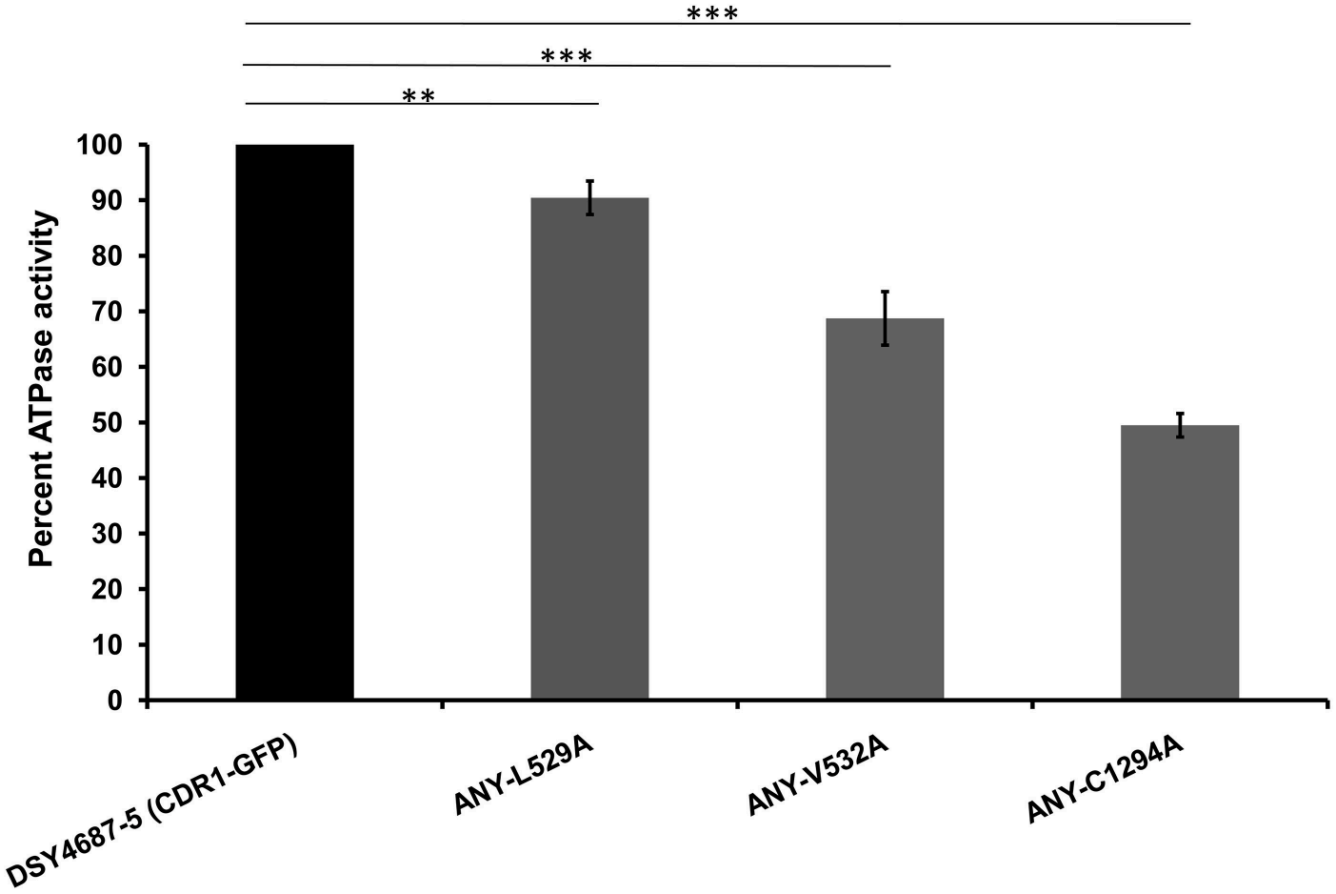
**Oligomycin-sensitive ATPase activity of DSY4687-5 and its mutant forms monitored by P_i_ released from the PM fractions of the strains**. Complete method described in the experimental section. Value of the WT strain (DSY4687-5) was kept at 100%. Background oligomycin-sensitive ATPase of the host has been subtracted from all the respective values. ATPase activities for the host (DSY4684) and WT (DSY4687-5) ranged between 15 and 20 nmoles Pi/mg of total PM protein/min and 60–75 nmoles Pi/mg of total PM protein/min, respectively. ^**^p ≤ 0.01; ^***^p ≤ 0.001.

R6G efflux, Nile red (NR) and [^3^H] FLC accumulation assays were performed with the WT and the constructed mutant variants to assess whether the enhanced drug susceptibility of the mutants coincided with the defect in substrate transport. Flow cytometry which was employed to measure the NR accumulation revealed lower accumulation of the fluorescent substrate in WT cells when compared to DSY4684. All three mutant strains, ANY-L529A, ANY-V532A, and ANY-C1294A, which were susceptible to drug(s), displayed intermediate levels of accumulation of NR (Figure 5A). R6G, another well-known fluorescent substrate of Cdr1p, was also used to monitor the efflux potential of the mutant variants. In accordance with the susceptibility profile for R6G, all the mutants showed a defect in transport of this fluorescent substrate (Figure 5B). The [^3^H] FLC accumulation assay corroborated the susceptibility profile of the mutants on FLC. While ANY-L529A, ANY-V532A showed a statistically significant defect in FLC transport, ANY-C1294A transport profile was unchanged as compared to WT. Together, the transport assays suggested that defect in the transport abilities of mutant variants of Cdr1p coincided with their antifungal susceptibilities. In spite of the fact that C1294A mutant was only partially resistant to R6G, it displayed decreased transport similar to that of the other two mutants. The oligomycin sensitive ATPase activity of the PM fractions of WT and mutant strains were checked to address whether the introduced mutations could affect the ATP catalysis cycle that could also impact substrates accumulation levels. Although ANY-L529A and ANY-V532A had statistically significant lower ATPase activities, this decrease (both strains retained over 65% activity of WT levels, Figure 6) is not considered as significant for altering substrate transport. This is also supported by the case of the ANY-C1294A variant which retained resistance to azoles and was only partially susceptible to R6G, even if exhibiting low ATPase activity (50% activity as compared to WT, Figure 6). Thus, any major role of low ATPase activity for the observed phenotypes could be excluded. Of note, the extensive kinetic analysis has revealed that the three investigated Cdr1p mutants were defective in substrate binding and not in ATP binding and catalysis when expressed in *S. cerevisiae* (Rawal et al., 2013).

### FK520 chemosensitizes Cdr1p overexpressing *C. albicans* cells to fluconazole

**Figure 7:**
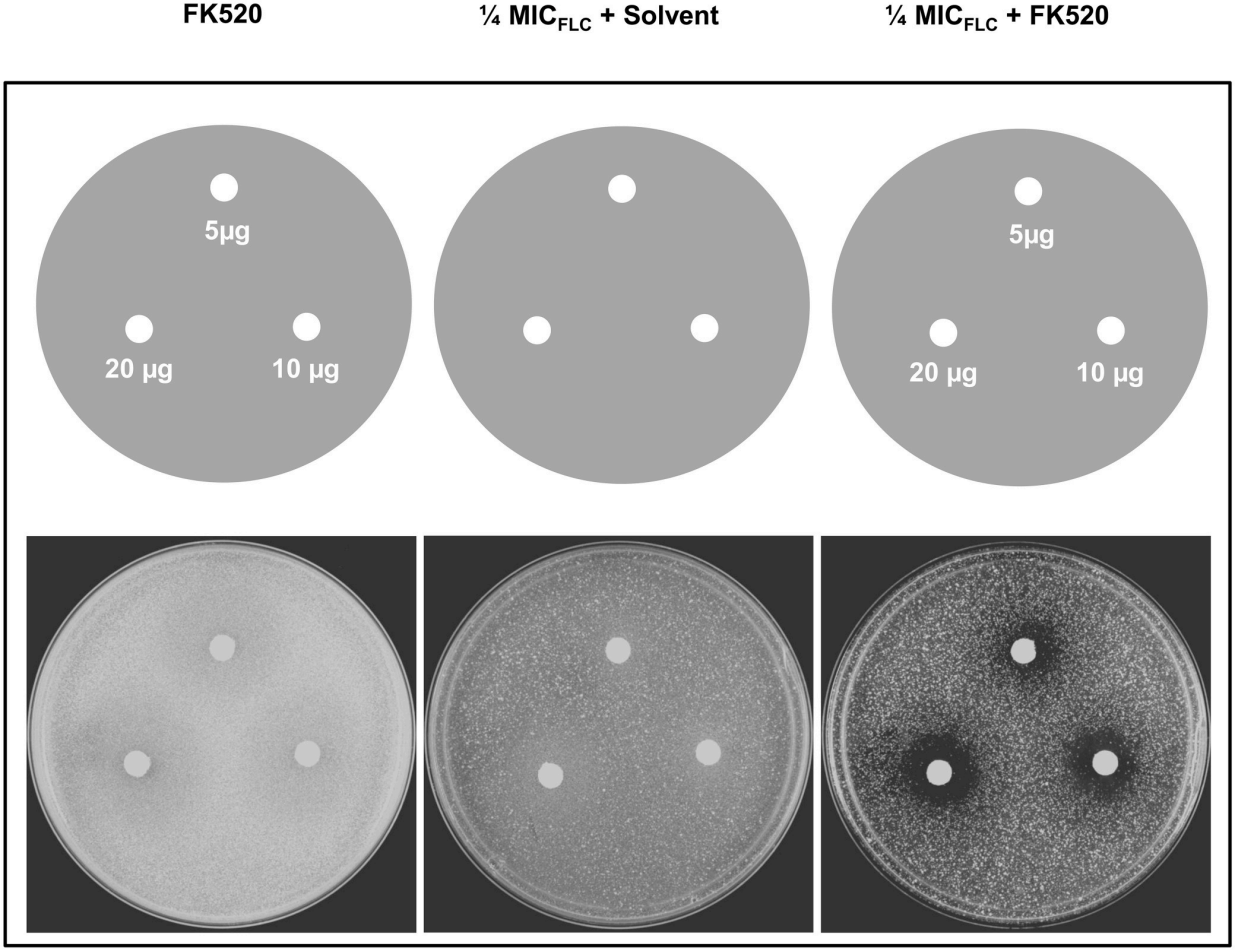
**Effect of FK520 on Cdr1p pump activity**. DSY4687-5 cells were analyzed by drug-diffusion assays on YEPD agar containing no FLC (left most panel) or FLC at ¼ MIC_FLC_ (right most panel). Indicated amounts of the inhibitor were spotted on each filter disk. In the middle panel, solvent for FK520 (ethanol) was placed on the disk as a solvent control.

We tested the ability of a calceneurin inhibitor FK520 to chemosensitize DSY4687-5 cells to FLC. Agar drug diffusion assay was utilized for this particular investigation. In order to exclude any growth interference as a result of toxic effects of the inhibitor itself, the assay was also carried out in the absence of FLC. Despite that there was a minor reduction in growth of the cells when exposed to only ¼ MIC_FLC_, it was observed that FK520 significantly enhanced the growth inhibition potential of FLC in the Cdr1p overexpressing system (Figure 7).

### MFS transporter Mdr1 could be functionally expressed in DSY4684

**Figure 8:**
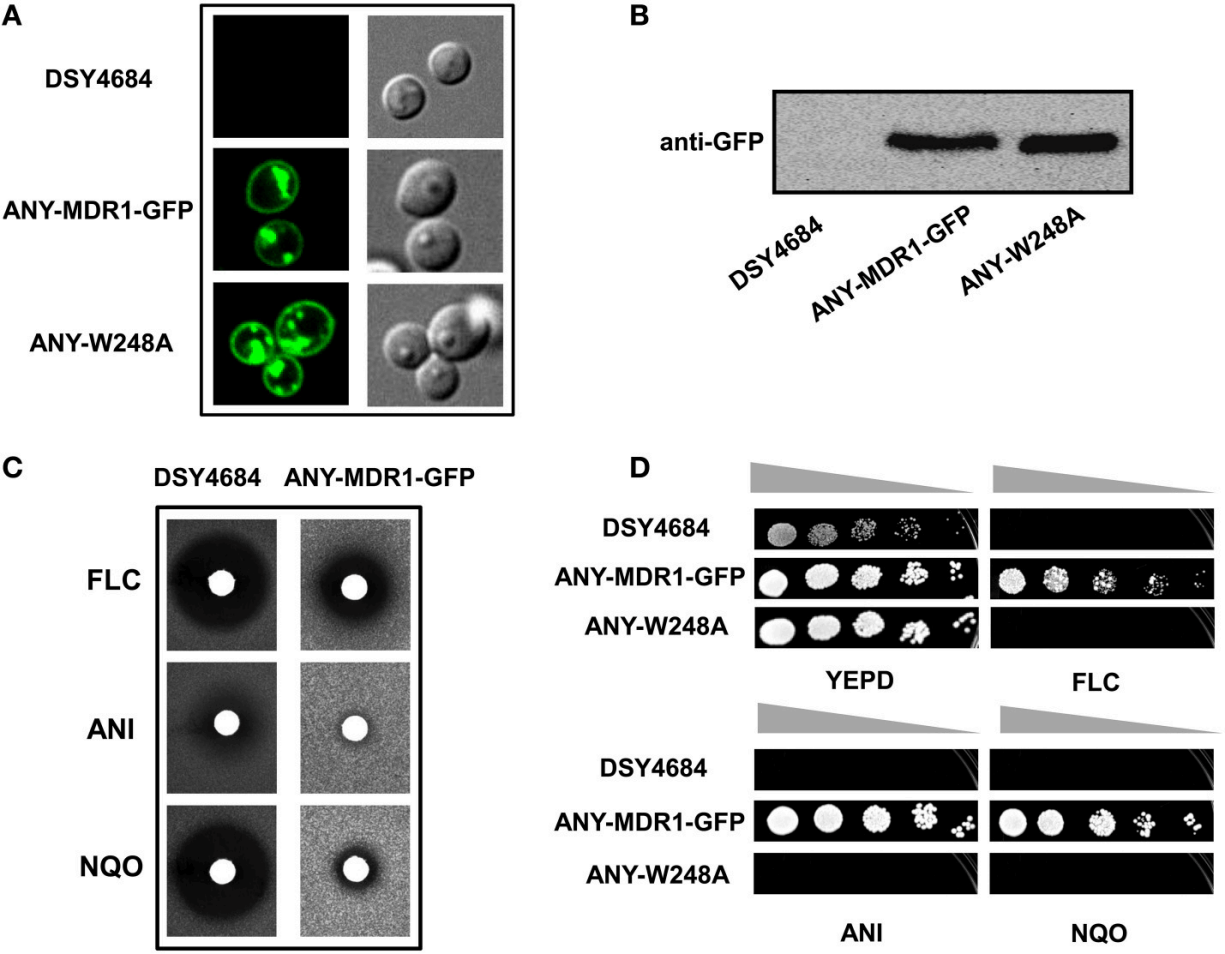
**The endogenous overexpression system for expression of a MFS superfamily protein**. *MDR1* with a C-terminal GFP tag was cloned into pDS1874 and overexpressed in DSY4684, the strain was designated as ANY-MDR1-GFP. **(A)** Confocal microscopy images of DSY4684, ANY-MDR1-GFP and its mutant. **(B)** Immunoblot analyses of their PM fractions (50 μg total protein) with anti-GFP monoclonal antibody. **(C)** Agar drug-diffusion assays of DSY4684 and ANY-MDR1-GFP cells. Susceptibility to the following xenobiotics were tested: Fluconazole (FLC; 10 μg), Anisomycin (ANI; 6 μg) and 4-nitroquinoline 1-oxide (NQO; 1 μg). **(D)** Drug resistance profiles of DSY4684, ANY-MDR1-GFP and its mutant variant determined by serial dilution assays. For serial dilution assays, the following drugs were used: ANI (4 μg/ml), FLC (4 μg/ml) and NQO (0.25 μg/ml).

We explored the newly developed overexpression system for the structure- function analyses of non-ABC membrane transporters. For this, we cloned the *C. albicans* MFS multidrug transporter protein *MDR1* with a GFP tag in pDS1874.After elimination of a BglII site present in *MDR1*, the expression plasmid was linearized by BglII and introduced in *CDR1* promoter of DSY4684. The resultant strain was designated ANY-MDR1-GFP. Confocal microscopy and immunoblot analyses confirmed the proper localization of Mdr1p to the PM (Figures 8A,B). Agar drug diffusion assay showed that overexpressed Mdr1-GFP was fully functional and could transport the three tested drug substrates [FLC, Nitroquinoline-oxide (NQO) and Anisomycin (ANS)] (Figure 8C). To further validate functionality, we also introduced a mutation at one crucial position (W248A) into Mdr1-GFP and overexpressed this mutant variant in similar background and the resulting strain was designated as ANY-W248A. Serial dilution assays on agar medium containing drugs revealed that this W248A mutant variant was susceptible to all tested drugs, as was observed earlier when overexpressed in a *S. cerevisiae* system (Pasrija et al., 2007; Figure 8D).

## Discussion

It is evident from the current literature that membrane efflux pumps pose a major impediment to effective antifungal therapy (White et al., 1998; Sanglard and Odds, 2002). Under such a scenario, it is quite imperative to have a thorough understanding of the structure and function of multidrug transporters. Thus, one of the foremost requirements is the need of an overexpression system which could drive constitutive and functional expression of the drug transporter proteins with minimum artifactual effects. Such a system is not only expected to facilitate the functional characterization of these transporters, but would also provide a platform for screening of their drug susbstrates and inhibitors/modulators.

During the past several years, *S. cerevisiae* has been successfully exploited as an overexpression system which provided useful insight into the structure-function aspects and substrate/inhibitor screening of drug transporter proteins of *C. albicans*. Despite such applications of overexpressing system of *S. cerevisiae* by several laboratories, concerns were raised pertaining to the functionality of *C. albicans* proteins in a heterologous background. This concern is even more relevant if one considers that the homologs of MDR transporters of *C. albicans* and *S. cerevisiae* follow different regulatory circuits for their expression. Additionally, since all drug efflux pumps are PM localized, the subtle differences in membrane composition and architecture between the two yeasts could also have crucial implications in protein insertion, integrity and function. Lipid environment for instance, impacts drug transport and ATPase functions of P-glycoprotein (P-gp) (Saeki et al., 1991; Urbatsch and Senior, 1995; Sharom, 1997). In particular, the phase state of lipid bilayer is crucial for ATPase activity of P-gp (Sharom, 1997). The membrane component, cholesterol, is also well known to affect ATPase activity and function of P-gp (Rothnie et al., 2001). The yeast membrane ergosterol, a substitute of cholesterol, can also affect the efflux functions of Pdr5p (Kaur and Bachhawat, 1999). Our group has earlier observed that lipid species such as ergosterol and sphingolipids are crucial for proper insertion of Cdr1p to PM and substrate efflux (Krishnamurthy, 1999; Pasrija et al., 2008). Notwithstanding, several studies related to Cdr1p/Cd2p and Mdr1p of *C. albicans* have provided very useful information related to their structure and function in a heterologous expression system, even if expression of heterologous proteins in *S. cerevisiae* can be associated with potential artifacts.

This study addressed these concerns by developing an endogenous overexpression system for *C. albicans* multidrug transporter proteins. We took advantage of an AR isolate containing a *TAC1* GOF mutation (Znaidi et al., 2007; Tsao et al., 2009). This strain possesses intrinsic high expression capacity of transporters. The deletion of major MDR pumps such as *CDR1, CDR2*, and *MDR1* made this strain amenable to expression of MDR variants. Stable integration and overexpression of a single gene copy under the control of a *CDR1* native promoter was made available. The expression plasmid integration can be performed by single site digestion (BglII) to enable integration into the genomic locus at the *CDR1* promoter.

This new endogenous overexpression system was validated by overexpressing a GFP tagged version of Cdr1p. The time course of GFP fluorescence in *C. albicans* cells expressing the fusion protein revealed that the protein was being properly trafficked to the cell surface. Our confocal microscopy and immunoblot analyses results showed that cells which were grown up to 8 hours had almost all their GFP fluorescence within the PM with only negligible amounts of trapped intracellular fluorescence. The overexpressed Cdr1p was fully functional since DSY4687-5 (CDR1-GFP) had significantly higher resistance to all the tested drugs as compared with the susceptible control strain lacking the known MDR pumps (DSY4684).

To further explore the suitability of the *C. albicans* overexpression system, we recreated earlier three well characterized crucial TMH mutants of Cdr1p in the endogenous system (Rawal et al., 2013). Our assays showed that two of the three mutants, L529A and V532A, behaved similarly as was the case when overexpressed in *S. cerevisiae.* Interestingly, the C1294A mutant showed a few contrasting phenotypes. For instance, even though R6G and NR transport in C1294A cells was impaired, the cells continued to display resistance to all the other tested drugs. [^3^H] FLC accumulation assay further confirmed that the mutant variant was specifically defective in R6G and NR transport.

Considering that Cdr1p possesses multiple overlapping mini-binding sites within the large binding cavity harboring common as well as specific residues for a particular substrate (Rawal et al., 2013), it is plausible that mutating C1294 to alanine might have specifically perturbed R6G and NR binding/transport. Contradictorily, it was not the case observed when the C1294A variant was overexpressed in *S. cerevisiae* as there it was found to be susceptible to all tested substrates.

The subtle differences in membrane environment between the two species could be responsible for this differential behavior of the mutant. Obviously, this aspect requires further investigation. Nevertheless, our results point toward distinctive behavior of membrane pump proteins when expressed in different environments.

The usefulness of this overexpression system for the identification and characterization of fungal pump inhibitors was evident from the fact that the calceneurin inhibitor FK520, which shows fungicidal synergism with azoles in the Cdr1p overexpressing *S. cerevisiae* system (Saini et al., 2005; Nim et al., 2014), also potentiated the growth inhibition of DSY4687-5 cells in the presence of FLC at much lower its MIC value.

In order to extend the application of this newly developed system for the analyses of other classes of multidrug transporters, we overexpressed a protein belonging to the MFS superfamily. For this purpose, Mdr1p of *C. albicans* was integrated in DSY4684. We also constructed a variant (Mdr1^W248A^) in the same background. Our results indicate that a non-ABC transport protein could also be functionally overexpressed in the PM of the host DSY4684. Expectedly, the cells overexpressing the Mdr1^W248A^ variant were susceptible to all tested drugs, which is consistent with earlier reports where W248 in TMH5 was identified as a crucial determinant of substrate recognition and transport in Mdr1p (Pasrija et al., 2007).

In conclusion, our results justifies the suitability and utility of this newly developed system for overexpression and functional characterization for a wide set of membrane proteins of *C. albicans*. Additionally, the lack of major endogenous MDR pumps and a *TAC1* mediated constitutive membrane protein overexpression provides a powerful platform to identify novel substrates and chemosensitizers. The system can still be optimized with the deletion of other MDR genes to serve as a valuable tool for studies on fungal MDR pumps.

## Author contributions

RP, DS, AB, and NKK conceived and designed the experiments. AB and NKK performed the experiments. RP, DS, AB, and NKK analyzed the data. RP and DS contributed reagents and materials. AB, RP, and DS contributed to writing of the manuscript.

## Funding

Work is supported by a grant to RP from the Department of Biotechnology: DBT No.BT/01/CEIB/10/III/02. Financial Support from Indo-Swiss Research grant (ISJRP grant No. 122_917) to DS and RP, for supporting NK's visit to CHUV, Lausanne is acknowledged. DS is supported by a Swiss National Research Foundation grant 31003A_146936.

### Conflict of interest statement

The authors declare that the research was conducted in the absence of any commercial or financial relationships that could be construed as a potential conflict of interest.
