# Supplementary material for: A New Endogenous Overexpression System of Multidrug Transporters of Candida albicans Suitable for Structural and Functional Studies
Source: Front Microbiol. 2016 Mar 3;7:261. doi: 10.3389/fmicb.2016.00261 (PMC4776216; doi:10.3389/fmicb.2016.00261)
Supplement: Supplementary file 1 [file Table1.DOCX]

**Supplementary table**

**Table S1**. List of plasmids used in the study.

| **S.No.** | **Plasmid name** | **Description** | **Source** |
| --- | --- | --- | --- |
| 1. | pDS1859 | Insertion of *CDR1* terminator in CIp10 | This study |
| 2. | pDS1866 | Insertion of *CDR1* promoter in pDS1859 | This study |
| 3. | pDS1869 | Insertion of *PDR5* terminator in pDS1866 | This study |
| 4. | pDS1874 | Insertion of CDR1-GFP in pDS1869 | This study |
| 5. | pAN-MDR1 | Insertion of MDR1-GFP in place of CDR1-GFP in pDS1874 | This study |
