# Supplementary material for: A New Endogenous Overexpression System of Multidrug Transporters of Candida albicans Suitable for Structural and Functional Studies
Source: Front Microbiol. 2016 Mar 3;7:261. doi: 10.3389/fmicb.2016.00261 (PMC4776216; doi:10.3389/fmicb.2016.00261)
Supplement: Supplementary file 2 [file Table2.DOCX]

**Supplementary table**

**Table S2**. List of oligonucleotides used in the study.

| **S.No.** | **Oligonucleotide** | **Sequence (5’-3’)** |
| --- | --- | --- |
| 1. | CDR1ter_SacI | GTTTGGAGCTCTTTTTCTTTCTAATTATAATTT |
| 2. | CDR1Ter_NotI | CATGGCGGCCGCTCAAACGTCTAAAATATAATAGAC |
| 3. | CDR1-Apa | AAAGCGGGCCCTCGTTACTCAATAAGTATTAATT |
| 4. | SpeKO3 | ACTGGTACTCCTAGTAAAGGTTTTTGTGTTACACCATATGTT |
| 5. | SpeKO5 | AACCTTTACTAGGAGTACCAGTGGAAAAGAATTCTTTTTGTT |
| 6. | CDR1-SpeI | GCGCAAACTAGTAATTTTTTTCTTTTTGACCTTT |
| 7. | CDR1/L529A-FP | GTAATGGGTCTCATAGCGTCGTCGGTCTTCTA |
| 8. | CDR1/L529A-RP | TAGAAGACCGACGACGCTATGAGACCCATTAC |
| 9. | CDR1/V532A-FP | CATATTGTCGTCGGCCTTCTATAATCTTAG |
| 10. | CDR1/V532A-RP | CTAAGATTATAGAAGGCCGACGACAATATG |
| 11. | CDR1/C1294A-FP | GTACCATAGCATTTTTCGCTTGGTATTATCCATTAG |
| 12. | CDR1/C1294A-RP | CTAATGGATAATACCAAGCGAAAAATGCTATGGTAC |
| 13. | MDR1/W248A-FP | GTTGGGTTAGCCGCTGCTAGTTTAGGTGCTG |
| 14. | MDR1/W248A-RP | CAGCACCTAAACTAGCAGCGGCTAACCCAAC |
