# Supplementary material for: A New Endogenous Overexpression System of Multidrug Transporters of Candida albicans Suitable for Structural and Functional Studies
Source: Front Microbiol. 2016 Mar 3;7:261. doi: 10.3389/fmicb.2016.00261 (PMC4776216; doi:10.3389/fmicb.2016.00261)
Supplement: Supplementary file 3 [file Image1.PDF]

Supplementary image

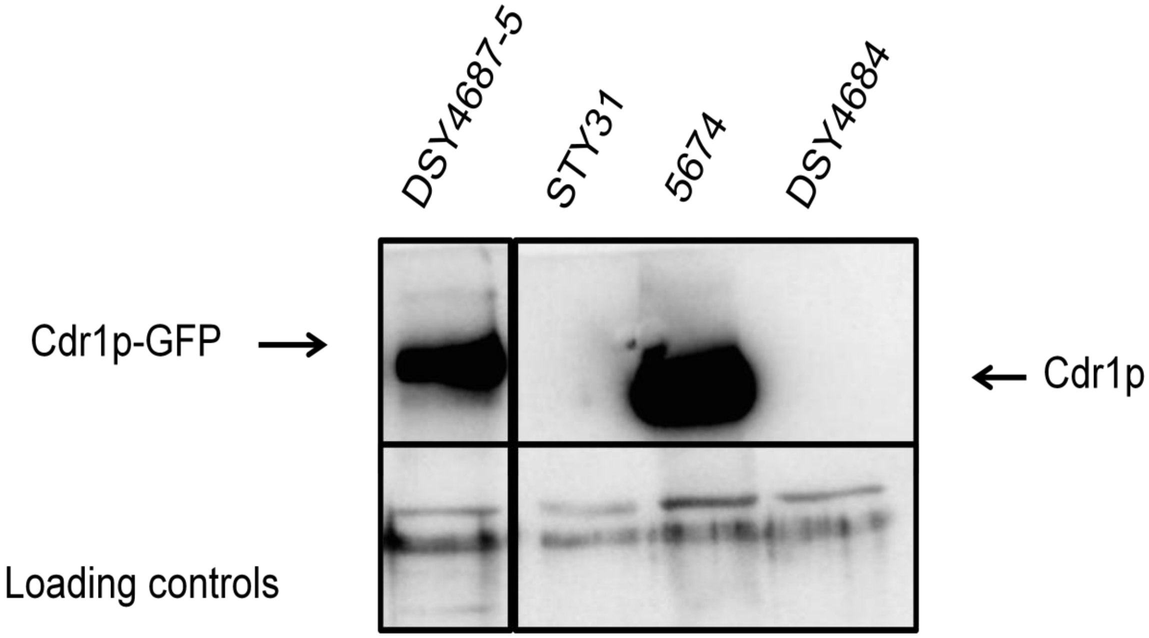

Figure S1: Immunodetection of Cdr1p in *C. albicans*. Strain designations are indicated and are described in Table 1 of the main text. The Cdr1p antibody produces Cdr1-specific signals and unspecific signals which were used as loading controls of each lane.
